# Tuning Pd-nanoparticle@MIL-101(Cr) Catalysts for Tandem Reductive Amination

**DOI:** 10.1007/s10562-017-2208-0

**Published:** 2017-11-27

**Authors:** Amanda E. Anderson, Christopher J. Baddeley, Paul A. Wright

**Affiliations:** EaStCHEM School of Chemistry, University of St Andrews, Purdie Building, North Haugh, St. Andrews, Fife KY16 9ST UK

**Keywords:** Pd@MIL-101, Tandem reductive amination, Kinetic modelling

## Abstract

**Abstract:**

The versatility of MOFs as highly porous Lewis acidic supports for precious metal nanoparticles has been exploited for one-pot tandem reductive amination catalysis. MIL-101(Cr) loaded with Pd nanoparticles ca. 3 nm in size at 0.2–1 wt% has been used to catalyse the reaction of 4′-fluoroacetophenone with benzylamine under 10 bar of H_2_ to give the secondary amine, 4′-fluoro-α-methyl-*N*-phenylmethylbenzenemethanamine. For the highest Pd loading, major hydrogenolysis of the secondary amine occurs in a second tandem reaction, but by changing the ratio of Pd to Lewis acidic Cr^3+^ active sites it is possible to tune the catalytic selectivity to the desired 2° amine product. An empirical kinetic analysis was performed to demonstrate this active site complementarity.

**Graphical Abstract:**

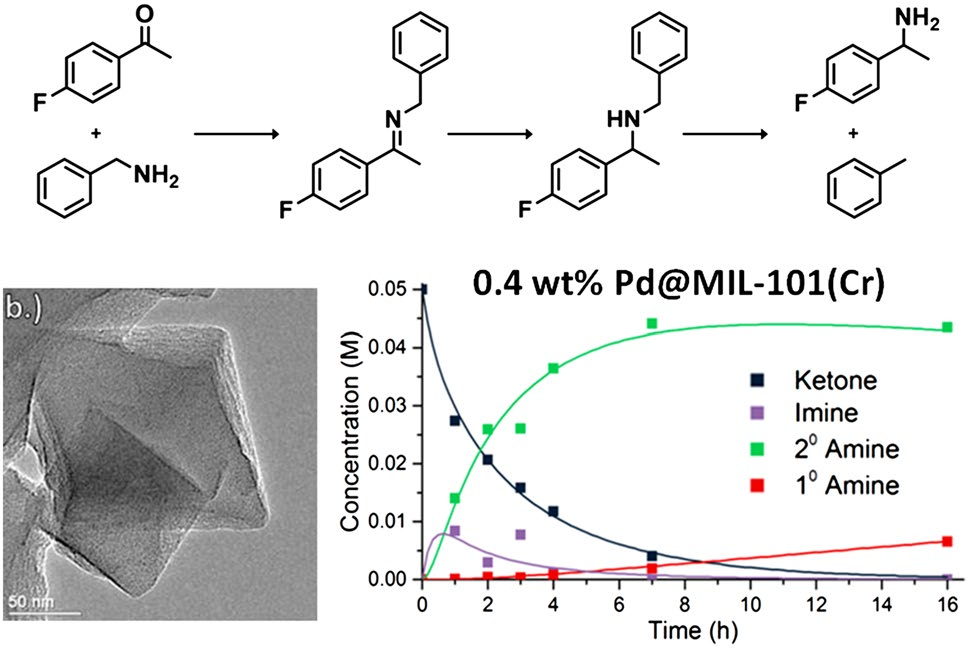

**Electronic supplementary material:**

The online version of this article (doi:10.1007/s10562-017-2208-0) contains supplementary material, which is available to authorized users.

## Introduction

Metal organic frameworks (MOFs) are highly porous crystalline materials synthesized from metal cation-based nodes and organic linkers. The enormous number of possible node and linker combinations makes the properties of these materials highly tunable and this has resulted in the MOF field receiving great attention in the past decade [1–4]. The high porosity of these frameworks, together with their ability to host catalytically active metal sites, has led to the development of many interesting catalytic materials [2]. As some MOFs contain mesopores of a few nanometres, they can encapsulate metal nanoparticles while allowing rapid transport of reactants and products through the pores. Loading MOFs with metal nanoparticles has been achieved by many methods ranging from gas-phase loading [5, 6] and solution deposition [7–9] to incipient wetness impregnation [10, 11].

**Fig. 1:**
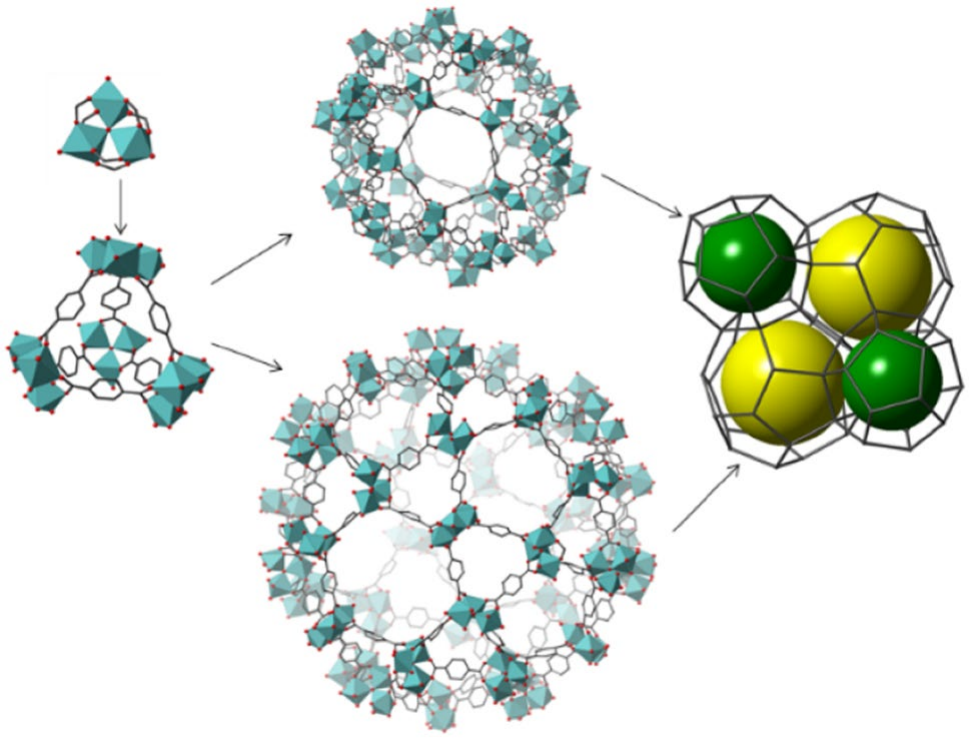
Schematic representation of MIL-101(Cr), showing the Cr_3_O(O_2_C–)_6_ cluster, the super-tetrahedral building units and the smaller (green spheres) and larger cages (yellow spheres)

The chromium terephthalate MOF, MIL-101(Cr) [12] has been widely studied as a catalyst because it possesses large pores, large three-dimensional porosity, good chemical and thermal stability and Lewis acidity [13]. Its structure is built from corner-sharing supertetrahedra arranged to give larger (3.4 nm) and smaller (2.9 nm) cages in an MTN arrangement, as seen in Fig. 1. MTN refers to the topology type for tetrahedrally connected nodes, denoted by the International Zeolite Association Structure Commission [14]. The mesoporous cages can be accessed through hexagonal and pentagonal windows with free diameters of 1.6 and 1.2 nm, respectively.

The pore size and chemical stability of MIL-101(Cr) make it amenable as a support for metal nanoparticles less than a few nm in size. As well as giving an active supported metal catalyst for reactions including oxidations and reductions [15, 16], the presence of Lewis acidic, coordinatively unsaturated, Cr^3+^ cations in the Cr_3_O(O_2_C–)_6_ trimers of MIL-101(Cr) enables bifunctional catalysts to be prepared. The use of bifunctional catalysts in one-pot procedures can eliminate the need for intermediate steps of separation and purification in multi-step reactions and catalysts comprising platinum or palladium nanoparticles supported on MIL-101(Cr) have been studied for reactions such as reductive aminations, where both Lewis acidity and hydrogenation activity is required.

**Scheme 1:**
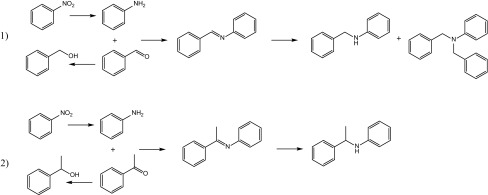
Reductive amination reactions that form secondary arylamines

Cirujano et al. [17] reported the reductive amination of nitrobenzene via aniline with either benzaldehyde or acetophenone (Scheme 1, reactions 1 and 2) over MIL-101(Cr) loaded with Pd by an incipient wetness technique, for which TEM indicates Pd nanoparticles ca. 5 nm in size are located on the external surface of the MIL-101(Cr) crystallites. The catalyst gave full conversions at 110 °C and 5 bar H_2_ after 6 h for reaction 1 and after 70 h for the more demanding reaction 2, with selectivities of 75 and 82% to the secondary amine.

Chen et al. [18] used Pd/Ag nanoparticles in MIL-101(Cr) for the same reactions, where the alloy nanoparticles were included by the ‘double solvents’ method of Aijaz et al. [19]. In this method the metal salt is dissolved in an aqueous solution while the MOF is dispersed in a non-polar solvent. Slowly adding an amount of aqueous solution less than the pore volume of the MOF allows the MOF to take up the metal salt within its hydrophilic pores by capillary action while the non-polar solvent minimizes the metal salt depositing on the surface of the MOF. This deposition technique leads to small and highly dispersed metal nanoparticles. The selectivity of the reaction to the desired secondary amine was then tuned by varying the Pd/Ag ratio in the nanoparticles. Using a Pd_2_Ag_1_@MIL-101(Cr) catalyst, 99% conversion was achieved in reaction **1** with 85% selectivity to the desired 2 amine, whereas for the more challenging reaction **2** the same catalyst achieved 65% conversion in 30 h with 71% selectivity. These results indicate the efficacy of the double solvents method to prepare active bifunctional and tunable catalysts.

**Scheme 2:**
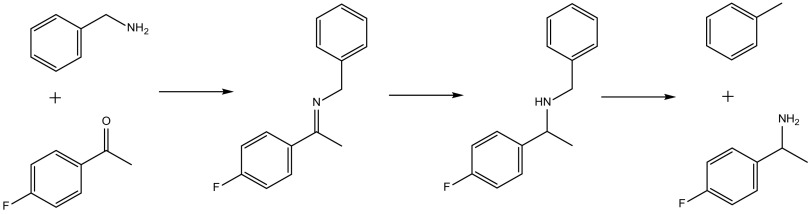
The reductive amination reaction studied in this work

Here, we report the catalytic performance of a series of Pd@MIL-101(Cr) catalysts prepared using the ‘double solvents’ method in a tandem reductive amination similar to those studied by Cirujano et al. and Chen et al. [18–20]. For our model reductive amination reaction we investigate the imine formation from 4′-fluoroacetophenone with benzylamine catalysed by the Lewis acid site in the MOF. We have previously shown that this reaction requires a Lewis acid catalyst of appreciable strength operating at elevated temperature to achieve high conversions [21], so that it provided a suitable first step for a reductive amination of the type we were interested in investigating. The imine then reacts on the palladium nanoparticle surface to reduce to a secondary amine in the presence of hydrogen. Under certain conditions, the palladium nanoparticles can catalyse hydrogenolysis of the 2° amine leading to decomposition into primary amine and toluene (Scheme 2). Here, we have varied the Pd nanoparticle loading to optimise the complementarity of the kinetics of the desired reactions of imine formation and hydrogenation in a one-pot reaction while minimising unwanted hydrogenation of the substrate ketone and subsequent hydrogenolysis of the secondary amine.

A fluorine-containing substrate was chosen to enable the progress of the reaction to be monitored readily by ^19^F NMR, and so to allow for kinetic modelling of the desired imine formation and hydrogenation and also the undesired hydrogenolysis. By matching the observed conversions by a series of differential equations that represent the reaction steps, it was possible to model the reactivity over the series of Pd@MIL-101(Cr) materials. We report that it is possible, with the help of the empirical kinetic model, to tune the selectivity of the reaction to the secondary amine by changing the ratio of metal nanoparticle active sites to Lewis acid active sites while still maintaining high catalytic activity. This shows that complementarity of the rates of reactions over the two types of active sites is an important parameter in order to achieve selective tandem catalysis.

## Experimental

### Catalyst Preparation

#### MIL-101(Cr)

MIL-101(Cr) was prepared via a modification of the procedure reported by Férey et al. [12]. CrCl_3_·6H_2_O (Fisions, 95%), terephthalic acid (Aldrich, 98%) and water were combined in a 1:1:400 molar ratio within a Teflon-lined stainless steel autoclave. The solution was mixed for 10 min, then sealed and heated to 220 °C for 8 h. Upon cooling to room temperature, the solution was centrifuged and the supernatant decanted. The solid was refluxed in ethanol for 24 h to remove unreacted terephthalic acid from the pores, then centrifuged and dried overnight in air at 70 °C.

#### 0.2-1.0 wt% Pd@MIL-101(Cr)

Palladium was deposited within MIL-101 using the ‘double solvent’ method [18–20]. The as-prepared MOF was activated at 150 °C under vacuum for 16 h. Then the MOF was combined with dry hexane and sonicated for 30 min to disperse the MOF crystals within the hexane solution. The desired amount of PdCl_2_ was dissolved in H_2_O to ensure filling of 35% of the available pore volume of the MOF and the PdCl_2_ solution was added dropwise to the MOF suspension while vigorously stirring. The solution was stirred for an additional 3 h, then the supernatant decanted. The solid was left to dry 24 h at room temperature then activated at 150 °C under vacuum for 16 h. The activated material was then reduced under a flow of 5% H_2_ in N_2_ for 2 h at 200 °C (after a ramp of 1.5 °C min^−1^) to produce the final 0.2–1.0 wt% Pd@MIL-101(Cr) material.

### Characterization

Powder XRD patterns were collected on STOE STADI/P diffractometers using Cu K_α1_ radiation (λ = 1.54056 Å). Nitrogen adsorption was performed using a Micromeritics Tristar II 3020 unit. Samples were outgassed at 150 °C overnight prior to analysis. Data was collected at 77 K. Elemental analysis was performed by Mikrolab Kolbe to determine Pd loadings. TEM images were obtained using a Jeol JEM 2011 HRTEM with an accelerating voltage of 200 kV. The samples were prepared by grinding the powder with acetone and depositing the resulting solution on a carbon-coated Cu grid. XPS was performed on a Scienta ESCA-300 with an Al K_α_ X-ray source (1486.7 eV).

### Catalysis Testing

In a typical reductive amination catalysis run, 5 mol% 0.2–1.0 wt% Pd@MIL-101(Cr) material (activated previously at 150 °C for 4 h under vacuum) was combined with dry hexane under a nitrogen atmosphere. 4′-Fluoroacetophenone and benzylamine (in a 1:2 molar ratio, to favour the imine reaction) were added to the mixture. The amount of catalyst was calculated using an estimate of the MOF formula unit [Cr_3_O((OH,(OH_2_)_2_)(O_2_CC_6_H_4_CO_2_)_3_)] molecular weight and the molar quantity of 4′-fluoroacetophenone used in the catalysis. Three reaction scales were used, with the smallest total amount in kinetic experiments and the largest in recycling experiments to ensure easy recovery of the catalyst for the next run. Typical reaction amounts for MIL-101(Cr) catalysts can be found in Table 1. For comparison, in a ‘standard’ run, 10 mg of 5 wt% Pd/C commercial catalyst was also used.

**Table 1:** Typical reaction amounts for each type of reductive amination catalysis run

Reaction type	MIL-101(Cr) (mg)	4′-Fluoro-acetophenone (ml)	Benzylamine (ml)	Solvent hexane (ml)
Kinetics	2	0.0071	0.0127	1
Standard	10	0.03	0.06	5
Recycling	20	0.07	0.13	10

The vials were sealed with caps containing septa, pierced with needles to allow access of H_2_ gas to the reaction suspensions, then loaded into a stainless steel autoclave and pressurised with H_2_ to 10 bar overpressure. The autoclave was placed in an oil bath and kept at the desired temperature for the duration of the reaction. The reaction was then quenched by placing the autoclave in water. After cooling and depressurising the autoclave was opened and the vials centrifuged to separate the catalyst from the reaction mixture. Minimal solvent loss was observed in each scale of reaction. Conversions and selectivities were determined using ^19^F NMR on a Bruker AV 400 machine and gas chromatography on a Thermo Trace GC Ultra with a Restek RTX-1 (30 m, 0.25 mm, 0.25 μm) capillary column was used to qualitatively observe other non-fluorinated species. ^19^F NMR was used to determine the conversions in the reductive amination catalysis. 4′-Fluoroacetophenone, *N*-[1-(4′-fluorophenyl)ethylidene]-benzenemethanamine (intermediate imine), 4′-fluoro-α-methyl-*N*-(phenylmethyl)-benzenemethanamine (2° amine), and 4′-fluoro-α-methyl-benzene methanamine (1° amine) were visible in the NMR spectra of products of reaction over MIL-101(Cr). Additionally, 1(4′-fluorophenyl)ethanol was observed over a 5%Pd/C catalyst. The NMR assignments are given in the Supplementary Information (SI: Table S1, Fig. S1). In addition, the research data (and materials) supporting this publication can be accessed at http://dx.doi.org/10.17630/5acc0b1b-145b-47f2-af0a-958b682e6742.

GC analysis showed that where a high concentration of the 1° amine is observed, the benzylamine is not present at double the concentration of 4′-fluoroacetophenone. For these reactions the toluene and 1° amine were not present in a 1:1 ratio, indicating the decomposition of the 2° amine is not the sole pathway to toluene formation and implying that another reaction is occurring on the Pd MNPs to convert benzylamine into toluene and ammonia. The consumption of benzylamine does not seem to compete with the imine formation reaction, though, because it is only observed at long reaction times, after consumption of the 4′-fluoroacetophenone, and then only for catalysts with higher Pd loadings.

To ensure the catalyst was not operating under diffusion limitation, a stir speed test was performed for the imine formation (the slower step of the tandem reaction). In this test, a series of imine formation reactions using MIL-101(Cr) was carried out at varying stir speeds for 6 h at 50 °C under N_2_. Five stir speeds were examined: 0, 100, 200, 400 and 600 rpm: all measured conversions were within experimental error. To maintain consistency throughout experiments, all catalytic reactions were performed with a stir speed of 400 rpm.

To determine whether the multifunctional materials were viable heterogeneous catalysts, recyclability experiments were performed. In each case the MOF is activated at 150 °C for 6 h under vacuum. The reaction is carried out for 16 h under 10 bar of H_2_. The catalyst is removed from the reaction solution by centrifugation, washed with ethanol, and reactivated for the next cycle.

To establish whether the catalysts act heterogeneously, a filtration test was performed on the 1.0 wt% Pd MIL-101(Cr) sample. In this experiment, 10 identical vials were prepared. All vials were put under reaction conditions and allowed to react for 3 h. After this time elapsed, 5 of the vials were removed and the reaction mixtures were centrifuged. The contents of one vial were analysed to find the conversion at 3 h and the other four vial contents were placed back in clean vials and subjected to reaction conditions again. At the end of every hour of reaction, two vials were removed—one with the catalyst still in the solution and one with the centrifuged solution mixture.

## Results and Discussion

### Catalyst Characterization

**Fig. 2:**
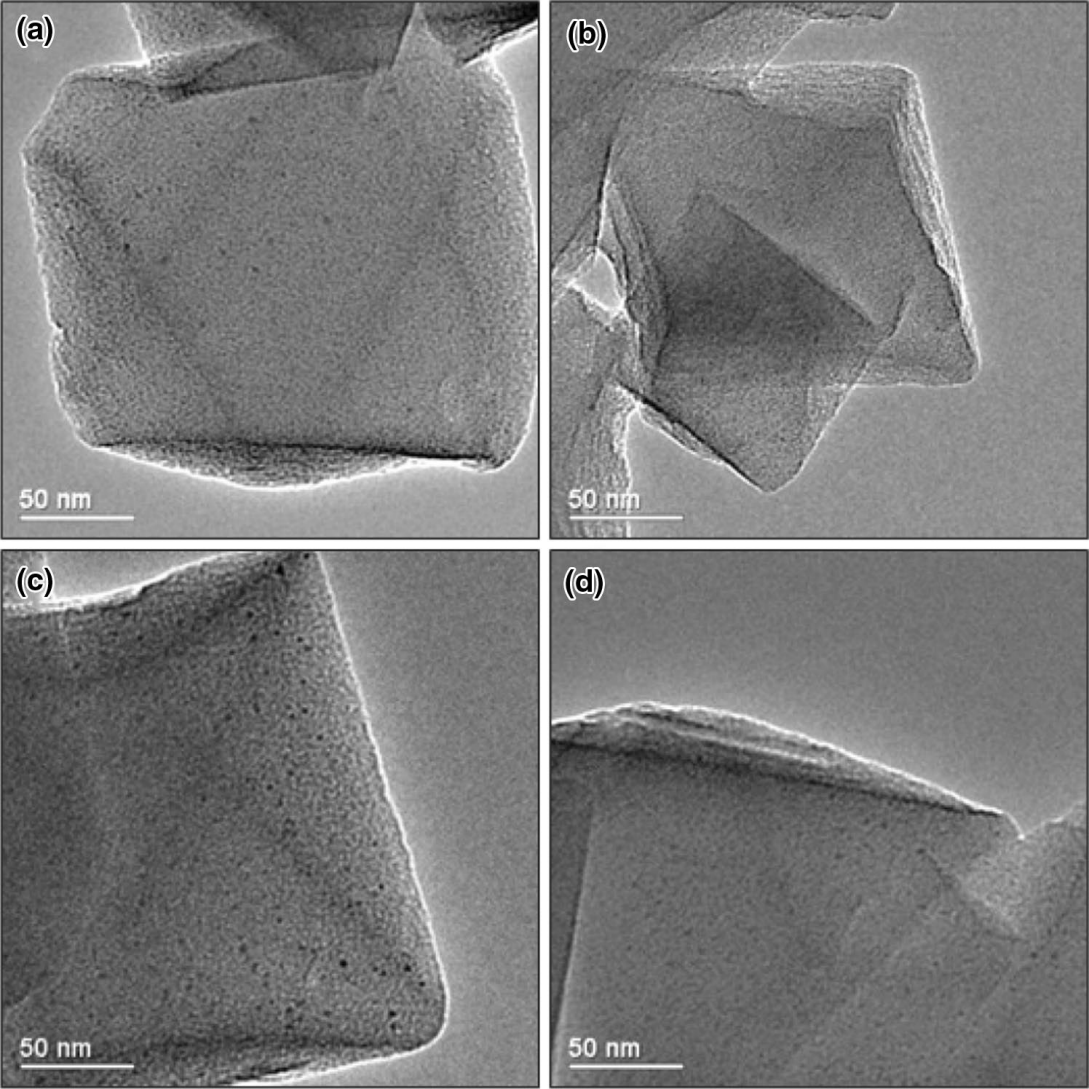
**a** 0.2 wt%; **b** 0.4 wt%; **c** 0.5 wt%; **d** 1.0 wt% Pd in MIL-101(Cr)

**Fig. 3:**
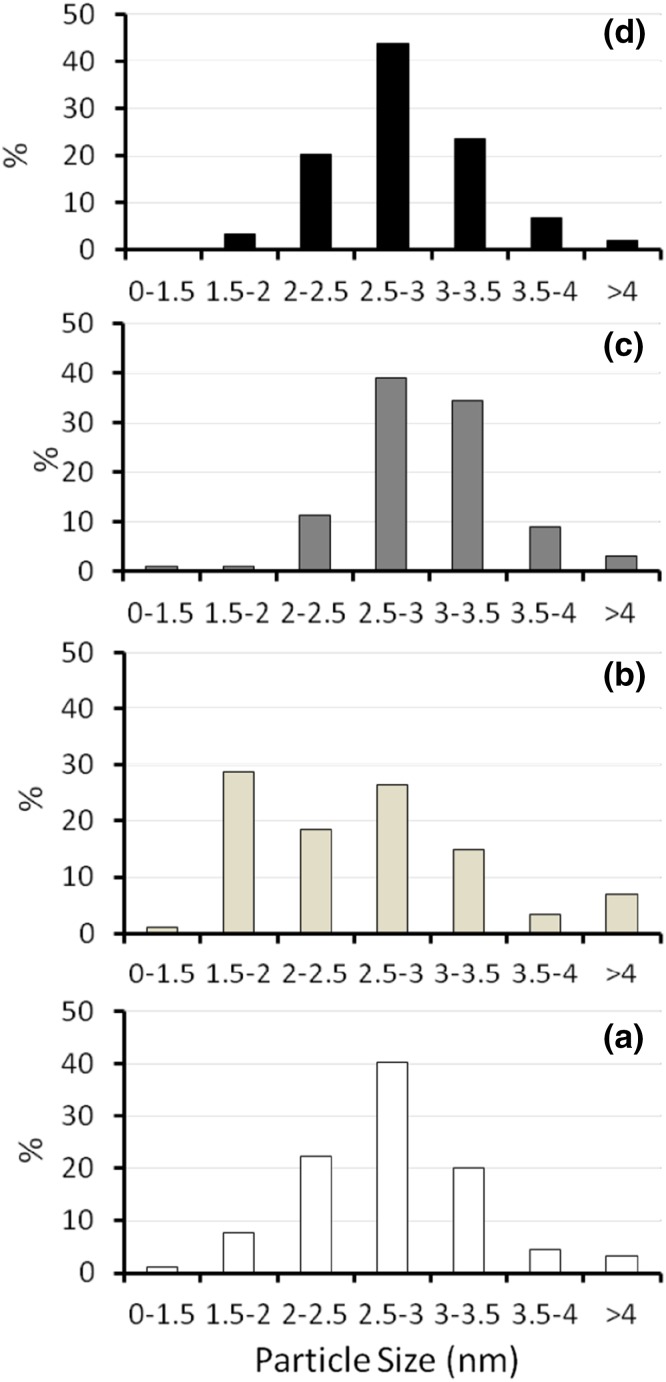
Measured Pd nanoparticle size distribution for **a** 0.2 wt%, **b** 0.4 wt%, **c** 0.5 wt% and **d** 1.0 wt% Pd on MIL-101(Cr) catalysts

X-ray powder diffraction (Fig. S2) of the as-prepared, ethanol-washed MIL-101(Cr) confirms its phase purity. Nitrogen adsorption at 77 K (Fig. S3) shows its surface area is 3013 m^2^ g^−1^, with a pore volume of 1.45 cm^3^ g^−1^. Palladium nanoparticles were successfully deposited within the pores of MIL-101(Cr) by the double solvents deposition method [18–20], which does not affect the crystallinity of the MIL-101(Cr) (Fig. S2). No X-ray reflections from Pd were observed in any of the materials with different Pd loadings which indicates the particles are less than a few nm in size. TEM images (Fig. 2) show octahedral MOF crystals and well-dispersed Pd nanoparticles with average sizes ranging from 2.6 to 2.9 nm and with narrow size distributions (Fig. 3). A summary of the physical properties of the Pd@MIL-101(Cr) materials is given in Table 2.

**Table 2:** Summary of physical properties of Pd@MIL-101(Cr) materials

Pd loading (wt%)	BET surface area (m^2^ g^−1^)	Pore volume (BJH adsorption) (cm^3^ g^−1^)	Average Pd size (e.s.d) (nm)
0	3013	1.45	n/a
0.2	3292	1.61	2.7 (± 0.6)
0.4	3061	1.52	2.6 (± 0.8)
0.5	3226	1.60	2.9 (± 0.6)
1.0	3253	1.63	2.8 (± 0.5)

Compared to the parent MIL-101(Cr) material, the MNP loaded materials show a slight increase in N_2_ adsorption (Table 2 and Fig. S3). This is probably because unreacted terephthalic acid is removed from the pores of MIL-101(Cr) during the double solvents procedure. The pore volume ranges from 1.45 cm^3^ g^−1^ for the parent MOF material to 1.63 cm^3^ g^−1^ for the 1.0 wt% Pd sample. Férey et al. [12] suggest that the pore volume varies between 1.5 and 1.9 cm^3^ g^−1^ as a result of different amounts of residual terephthalic acid in the pore system. Upon changing the Pd loading, the average Pd nanoparticle size remains very similar, allowing comparison of the activity and selectivity of the different Pd loaded catalysts as the main parameter changing through the materials is the overall number of nanoparticles and not their size. The main influence on the activity and selectivity in tandem reactions is the ratio of Pd nanoparticle active sites to Lewis acid active sites within the MOF. Notably, XPS analysis on the Pd@MIL-101(Cr) materials gave no signal for Pd, even after extended acquisition times, while Cr, C and O of the MOF were readily detected. This indicates very low levels of Pd within the XPS analysis depth of a few nm and no surface aggregation (Fig. S4).

### Catalytic Performance

The reductive amination reaction of Scheme 2 was used as a model reaction to tune selectivity and activity towards the desired 2° amine product. The first step is the condensation of 4′-fluoroacetophenone and benzylamine facilitated to form an imine, which is hydrogenated on the Pd nanoparticle to the desired secondary amine. Under certain conditions, the Pd nanoparticles can catalyse hydrogenolysis of the 2° amine leading to decomposition into undesired 1° amine and toluene, which reaction type was not observed in previous similar reductive aminations [17, 18]. The aim was to minimize the decomposition of the desired product while maximising the rates of the other reactions to obtain high activity and selectivity towards the 2° amine.

Starting with 1.0 wt% Pd@MIL-101(Cr) at 50 °C (Table 3, entry 1) full conversion is not achieved after 16 h of reaction. The selectivity is 9.1% imine, 90.7% 2° amine and less than 1% 1° amine.

**Table 3:** Catalysis results for Pd loaded MIL-101(Cr) at 16 h, 5 mol% MOF catalyst, with other experiments for comparison

Entry	Pd loading (wt%)	Temp. (°C)	Conversion (%)	Selectivity (%)		
				Imine	2° Amine	1° Amine
1	Control	50	1.0	100	0	0
2	1.0	50	65.1	9.1	90.7	0.2
3	1.0	90	100	0	8	91.2
4	0.5	90	95.2	0	75.3	24.7
5	0.4	90	100	0	86.8	13.2
6	0.2	90	100	0	89.9	10.1
7	0.0	90	78.0	100	0	0
8	Pd@C^a^	90	100	0	28.0^b^	0

Reaction conditions: 5 mol% MOF catalyst; 1 MPa H_2_ for 16 h. Conversions determined by ^19^F NMR based on consumption of 4′-fluoroacetophenone

^a^5% Pd supported on C

^b^Remaining 72% is the secondary alcohol 1(4′-fluorophenyl)ethanol

**Fig. 4:**
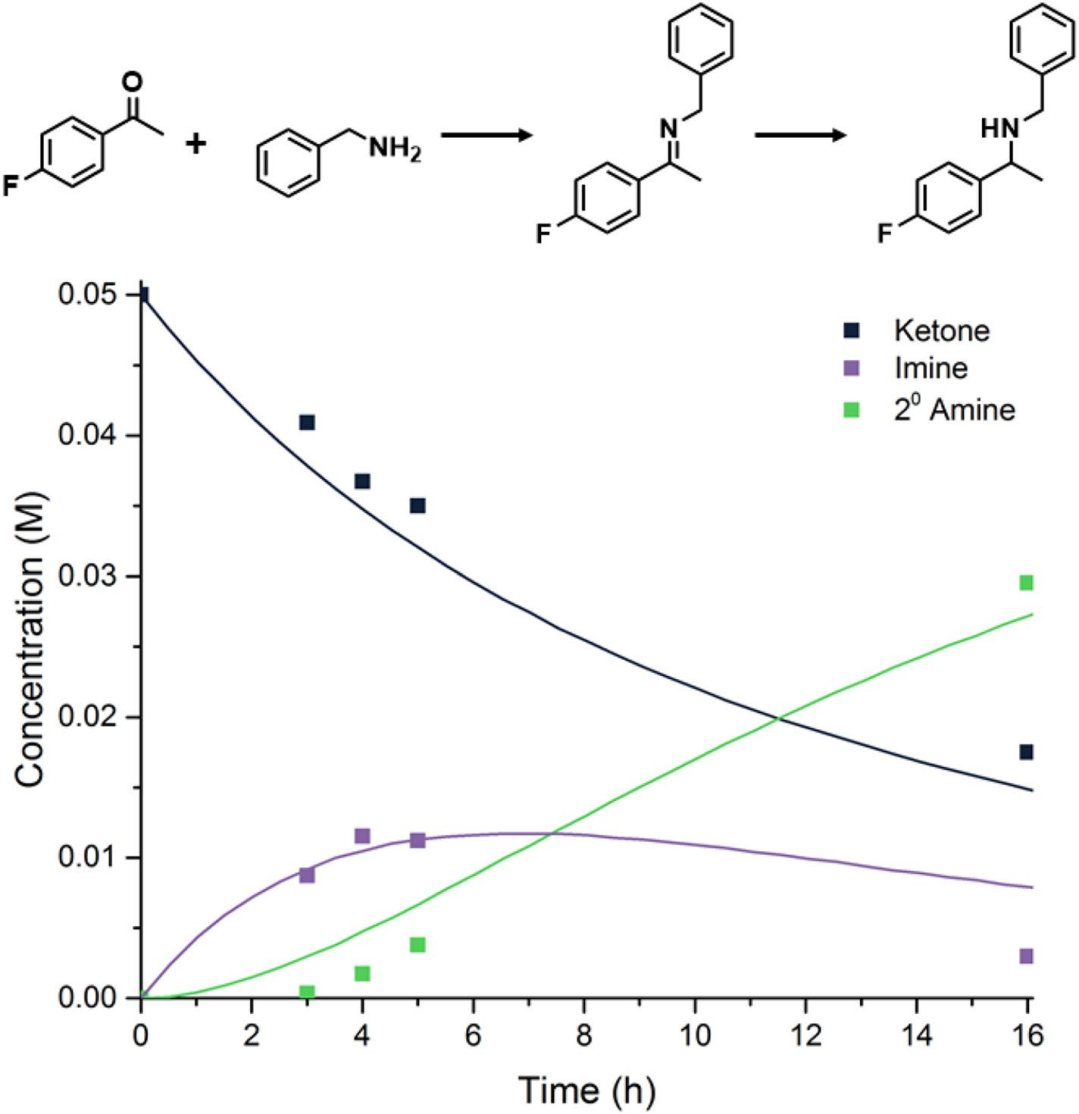
Reaction of 4-fluoroacetophenone with benzylamine under 10 bar H_2_ at 50 °C over 1%Pd@MIL-101(Cr) (5 mol% MOF catalyst)

The reaction conversion was also determined after 3, 4 and 5 h to investigate the initial steps of the reaction. Figure 4 shows the reaction progression. High selectivity to the desired 2° amine is obtained but only 65% of the starting 4′-fluoroacetophenone has reacted after 16 h, so full consumption of 4′-fluoroacetophenone would take too long to be useful at this temperature.

Removal of the catalyst after 3 h stopped both imine formation and imine hydrogenation, showing the catalysis was fully heterogeneous (Fig. S5) and that reaction is very slow without a catalyst. Recycling showed that Pd MIL-101(Cr) can be used three times under these conditions with minimal loss of activity and selectivity (Table 4). There is no loss of crystallinity of the MOF (Fig. S6) and TEM images show that after catalysis the Pd nanoparticles are still well dispersed throughout the MOF support (Fig. S7) although their size has increased slightly. After the third recycle, 80% of the MNP that were within the MIL-101(Cr) cage size range remain in this range.

**Table 4:** Recyclability of the 1 wt% Pd@MIL-101(Cr) at 50 °C

Run	Conversion (%)	Selectivity (%)	
		Imine	2° Amine
1	65	10	90
2	67	8	92
3	59	9	91

Increasing the temperature to 90 °C greatly increases the conversion over the same catalyst (Table 3, entry 2), giving full conversion after the same time period. However, the increase in temperature leads to a change in selectivity and the decomposition route is the main reaction pathway (Scheme 2). In the experiments which show the undesired 1° amine as the selective product we also observe the decomposition of benzylamine into toluene (and ammonia). This indicates an increased activity of the Pd nanoparticles at higher temperatures and the imine functionality is no longer hydrogenated selectively.

To optimise the Pd on MIL-101(Cr) catalyst three samples were prepared with lower Pd contents: 0.5, 0.4, and 0.2 wt%. As the ratio of Pd active sites to Lewis acid active sites is reduced by decreasing the Pd loading, a shift of selectivity in the catalysis is observed. Comparing the 0.5 wt% Pd loading to double this amount we observe a drastic increase in selectivity to the 2° amine to 75% (Table 3, entries 2 and 3). By decreasing the Pd loading to 0.4 and 0.2 wt%, the selectivity is further increased, to 87 and 90% respectively. This demonstrates that for the 16 h reaction, an almost complete switch of selectivity results, from 1° amine at high loading to 2° amine at low loading. As the Pd MNPs within each material possess approximately the same size and distribution, the difference in the samples is the ratio between the Lewis acid active sites and Pd active sites. As the number of Pd sites goes down, the selectivity towards the desired product increases.

If MIL-101(Cr) without Pd is used as a catalyst, only the Lewis acid catalysed imine formation is observed, with a lower conversion of 78% (Table 3). This indicates that subsequent reaction of the imine in the presence of Pd accelerates the observed conversion of the reactant ketone. Furthermore, if 5 wt% Pd supported on C is used in place of the Pd@MIL-101(Cr), complete conversion is achieved, but selectivity is mainly (72%) to 1(4′-fluorophenyl)ethanol, via hydrogenation of the reactant ketone, showing the requirement for the acidic function for the tandem reaction.

**Scheme 3:**
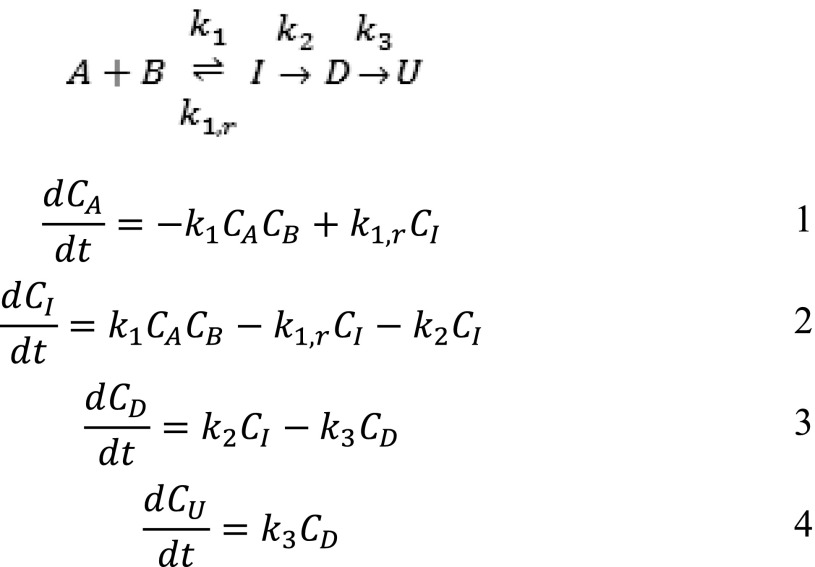
Kinetic scheme and differential equations used in MATLAB modelling of concentrations, C (*A* 4′-fluoroacetophenone, *B* benzylamine, *I* imine, *D* ‘desired’ secondary amine, *U* ‘undesired’ primary amine)

Insight into the selectivity towards the desired product was achieved by performing a kinetic investigation. For all four Pd-loaded catalysts the reaction progress was monitored in time and the concentration–time data matched using the program MATLAB (Mathworks Inc.) for the equilibrium of Scheme 3 and including the rate equations 1–4 given in Scheme 3, according to the reaction shown in Scheme 2.

**Fig. 5:**
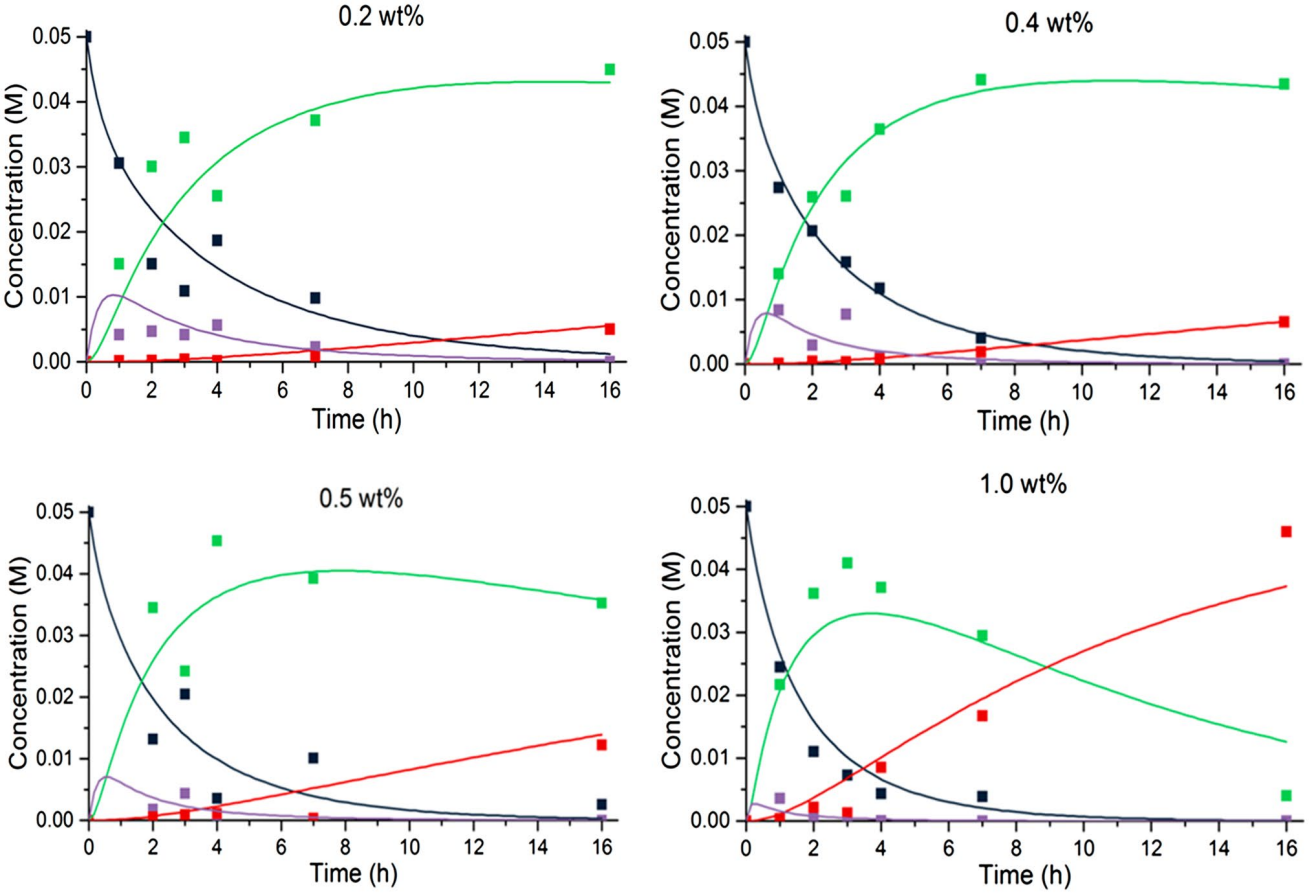
Pd@MIL-101(Cr) (0.2–1 wt% Pd) kinetic results at 90 °C compared with MATLAB numerical model. In each plot black squares represent the ketone, purple squares the imine, green squares the secondary amine and red squares the primary amine. Matched rate constants for each Pd loading are given in Table 5

The imine formation was modelled as a reversible reaction where the Lewis acid site can both form and decompose the imine. Attempts to fit the data by making the reverse of the imine formation step bimolecular (with H_2_O as a reactant) did not give an improved fit over a first order reaction, so it was modelled as the latter for simplicity. The two reactions that take place on the Pd surface (hydrogenation and hydrogenolysis) are modelled as first order reactions. This simplistic model fits the data well enough to visualize trends relating to activity and selectivity of the catalysis. By changing the rate constants for each reaction, the change in Pd site to Lewis acid site ratio could be investigated. The reaction rate constants for both the forward and reverse imine reactions are kept at the same value for each of the materials as the number of Lewis acid sites remains approximately the same. Figure 5 shows the model to experimental fits for each sample. The overall reaction rate constants determined to be a reasonable fit of the experimental data are shown in Table 5.

**Table 5:** Matched rate constants at 90 °C for Scheme 2 for each Pd-loaded MIL-101(Cr) catalyst

Entry	Pd loading (wt%)	k_1_ (M^−1^ h^−1^)	k_1,r_ (h^−1^)	k_2_ (h^−1^)	k_3_ (h^−1^)
1	0.2	8	1	1.1	0.01
2	0.4	8	1	2	0.011
3	0.5	8	1	2.5	0.025
4	1.0	8	1	10	0.1

The reaction rate constants for the Pd catalysed reactions, k_2_ and k_3_, are correlated with the number of Pd sites which varies with the metal loading. The reaction rate constant is a pseudo-constant because it must include a variable which changes with the number of active sites within the catalyst. Since the Pd particles stay approximately the same size, the Pd surface area should increase linearly with Pd loading. The reaction rate constants that describe the conversions catalysed by Pd MNPs increase roughly 10 times with a five-fold increase of the amount of Pd (0.2–1.0 wt%), in approximate agreement with the increase in Pd surface area. Comparing the models we find that the 0.4 wt% Pd MIL-101(Cr) sample achieves the highest conversion of the desired 2° amine in 7 h while keeping the 1° amine concentration low.

The 1.0 wt% Pd@MIL-101(Cr) sample was tested in a hot filtration reaction to determine the heterogeneity of the system. After 1 h at 90 °C, the catalyst was removed from the reaction solution and the supernatant was placed back under reaction conditions. Figure S8 shows that upon removal of the solid catalyst, no reaction occurs for any of the reactions in the multi-step tandem catalysis. This indicates both the Lewis acid active sites and the palladium metal active sites catalyse their respective reactions heterogeneously and no leaching of catalytically active material seems to occur. The confirmation of crystalline MOF after catalysis can be seen in the XRD patterns in Fig. S9.

TEM images of the catalyst before and after use showed small Pd MNPs within crystalline MIL-101(Cr) supports, seen in Fig. S10. The average MNP size increased from 2.8 to 3 nm, still well within the MIL-101(Cr) cage size. Recycling at elevated temperature was more difficult than at 50 °C, possibly due to the increased activity of the Pd MNPs at the higher temperature. The recyclability test was at 1 h to keep conversion low, thus being able to have a better assessment of catalyst degradation. The quenching of the reaction was performed by placing the hot autoclave into a water bath for 5 min before opening, although it is likely that the solutions remained warm. Upon recycle, while the conversion of ketone remained approximately constant at 38%, there was a decrease in selectivity towards the 2° amine (65 cf. 75%) because the amount of imine was higher. At these higher temperatures, it is likely that the Pd MNPs may have partially oxidised upon opening the autoclave, leading to a decrease in activity. To properly assess the reusability of this material under these conditions, the catalyst workup procedure between runs should be performed under inert conditions, minimizing contact of the Pd MNPs with air.

During the kinetic analysis, we attempted to measure the rate constants for the reversible imine formation directly. The reaction was run over MIL-101(Cr) at 10 bar H_2_ and 90 °C. A full analysis gave a good fit to the reaction progress using the rate constants k_1_ = 2.1 M^−1^h^−1^ and k_1,r_ = 0.03 h^−1^, modelling the reverse reaction as first order (Fig. S11). However, when these values were included in the MATLAB simulation of the tandem reaction over 1% Pd@MIL-101(Cr) in 10 bar H_2_ at 90 °C it was not possible to match the data because the rate of ketone consumption is much faster than predicted. Furthermore, replacing H_2_ with Ar over MIL-101(Cr) reduces the reaction rate further (Table S2). Using Pd@MIL-101(Cr) under Ar in place of MIL-101(Cr) slightly increased the rate but not back to that observed for MIL-101(Cr)/H_2_. These results indicate that H_2_ and Pd play complementary roles even in the imine formation step.

Comparing the catalysts tested in this reaction to similar published reactions [16, 17] it should be noted that fluorinated species were used in this work and benzylamine used in place of aniline. Nevertheless, compared with a similar reductive amination performed by Cirujano et al. [17], the reaction in this work proceeds significantly faster while obtaining similar selectivity, possibly due to the tuning of the ratio of active sites. Additionally, in contrast to similar multifunctional reactions performed by Chen et al. with Pd/Ag nanoparticles in MIL-101(Cr) [18], it is shown that the second metal is not necessary to tune selectivity in our reaction, but that in this case lowering the overall amount of active metal gives the same effect.

## Conclusions

Pd nanoparticles embedded in MIL-101(Cr) with metal loadings ranging from 0.2 to 1.0 wt% Pd were successfully prepared and compared for activity and selectivity in a multi-tandem reaction. The bifunctional catalyst uses both the Lewis acidity of the MOF support and the catalytic hydrogenation abilities of the Pd nanoparticles. Tuning the ratio of active sites by changing the Pd loading within the MOF was found to have a significant effect on the selectivity in the reductive amination reaction tested. The kinetic profiles have been simulated with a numerical solution of a series of differential equations. Higher metal loadings led to a significant amount of undesired 1° amine due to decomposition of the desired 2° amine product over the larger number of Pd sites. By reducing the overall number of Pd sites compared to Lewis acidic sites higher selectivity was achieved, with the 0.4 wt% Pd MIL-101 giving nearly 90% of the desired 2° amine after 7 h. This shows that activity and selectivity can be altered within multifunctional MOF materials by simply altering the ratio of metal nanoparticle active sites with respect to the Lewis acid active sites. Additionally, the materials were reusable and maintained crystallinity and small highly dispersed nanoparticles after reaction.

## Electronic supplementary material

Below is the link to the electronic supplementary material.

Supplementary material 1 (DOCX 1081 KB)
