# Supplementary material for: Tuning Pd-nanoparticle@MIL-101(Cr) Catalysts for Tandem Reductive Amination
Source: Catal Letters. 2017 Nov 27;148(1):154–63. doi: 10.1007/s10562-017-2208-0 (PMC6566290; doi:10.1007/s10562-017-2208-0)
Supplement: Supplementary file 1 — Supplementary material 1 (DOCX 1081 KB) [file 10562_2017_2208_MOESM1_ESM.docx]

**Supplementary Information**

**Tuning Pd-nanoparticle@MIL-101(Cr) Catalysts for Tandem Reductive Amination**

Amanda E. Anderson^1^ · Christopher J. Baddeley^1^ · Paul A. Wright^1,*^

^1^ EaStCHEM School of Chemistry, University of St Andrews, Purdie Building, North Haugh, St. Andrews Fife K16 9ST

Amanda E. Anderson [aea6@st-andrews.ac.uk](mailto:aea6@st-andrews.ac.uk)

Christopher J. Baddeley [cjb14@st-andrews.ac.uk](mailto:cjb14@st-andrews.ac.uk)

Paul A. Wright [paw2@st-andrews.ac.uk](mailto:paw2@st-andrews.ac.uk)

**Table S1** ^19^F NMR Peak Assignments for Fluorinated Species in the Reductive Amination Reaction

| Molecule | ^19^F NMR shift (ppm) |
| --- | --- |
| 4’-Fluoroacetophenone | -106.3 |
| Imine | -112.0 |
| 2° Amine | -115.6 |
| 1° Amine | -116.3 |


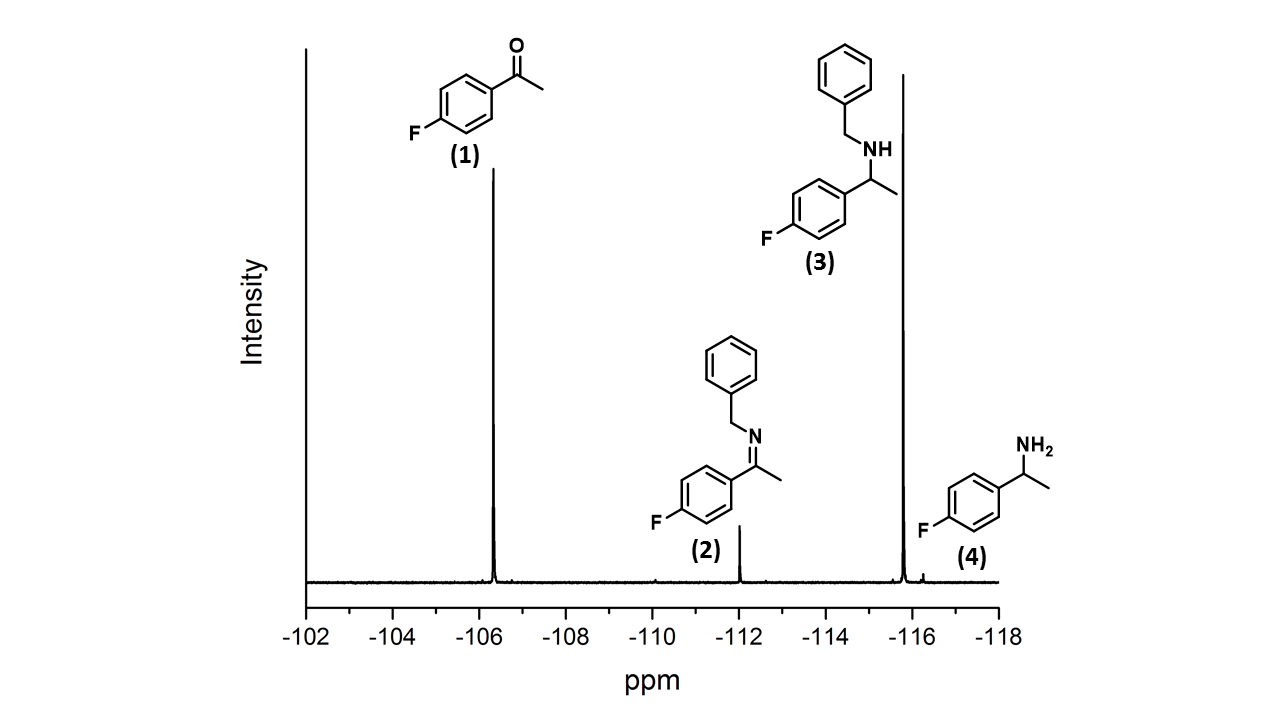


**Figure S1** Representative ^19^F NMR from a reductive amination catalysis experiment.


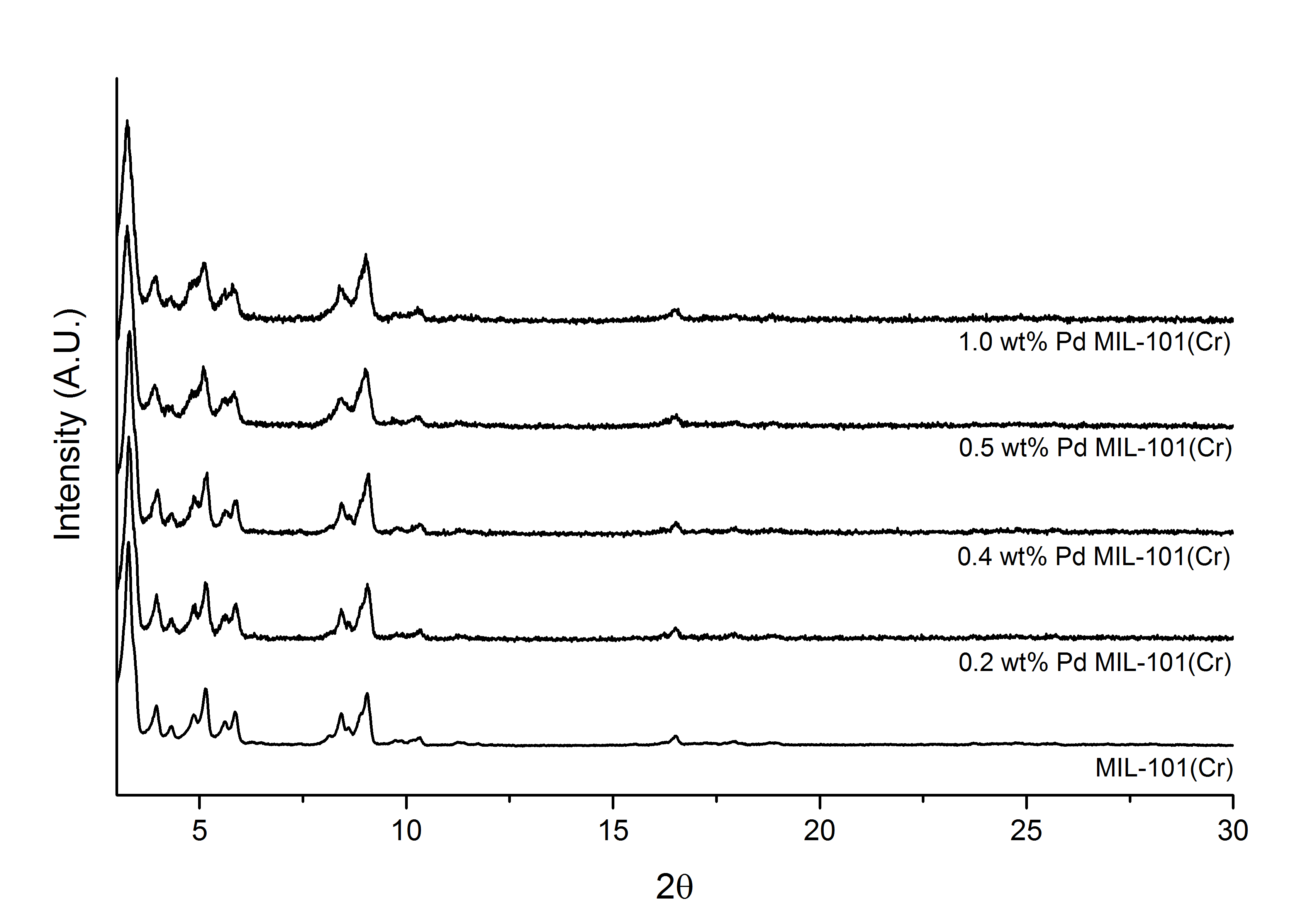


**Figure S2** Powder XRD patterns for the four Pd-loaded MIL-101(Cr) materials and the parent MIL-101(Cr) support before loading


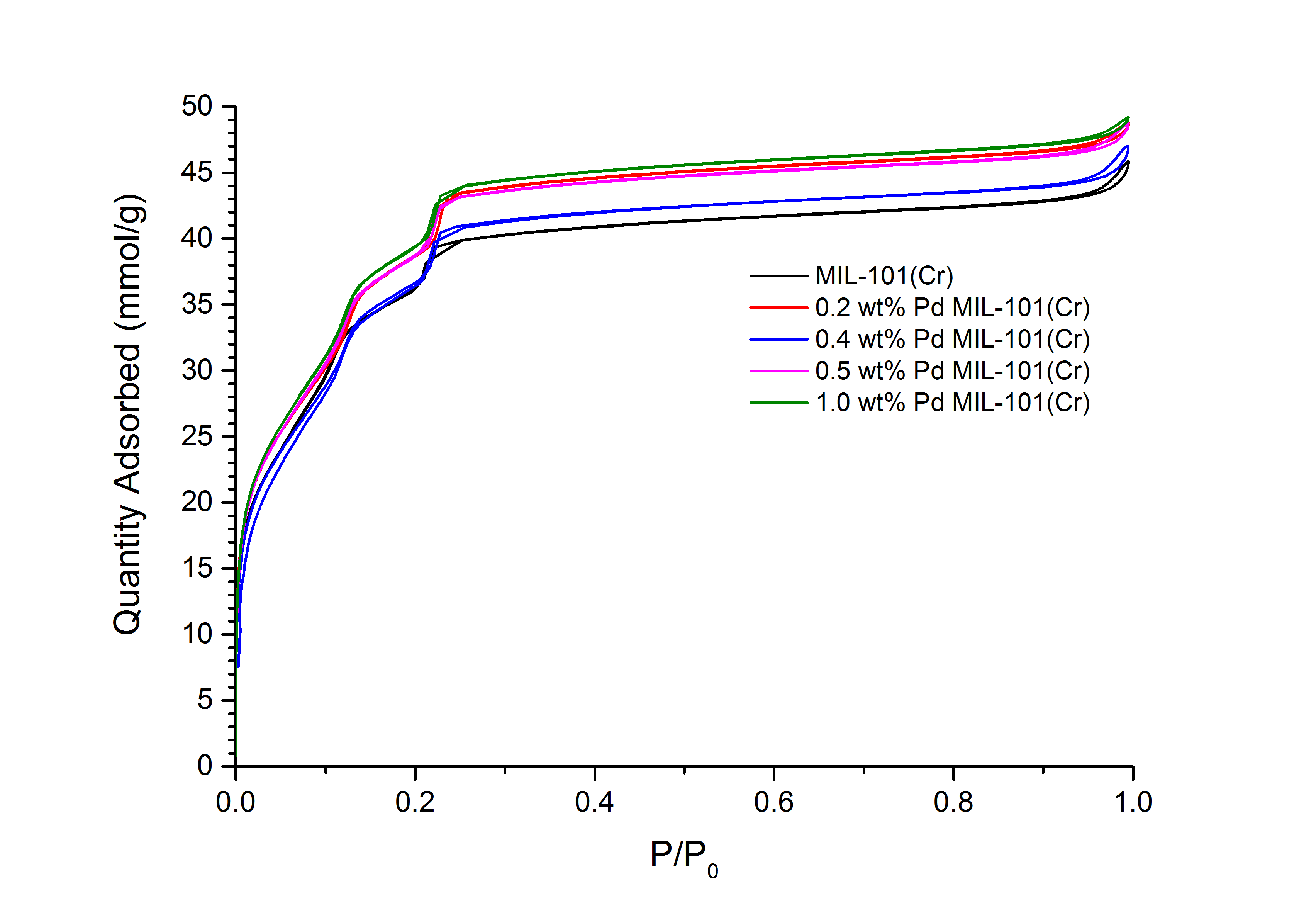


**Figure S3** N_2_ adsorption isotherms at 77 K on parent and Pd-loaded MIL-101(Cr) materials


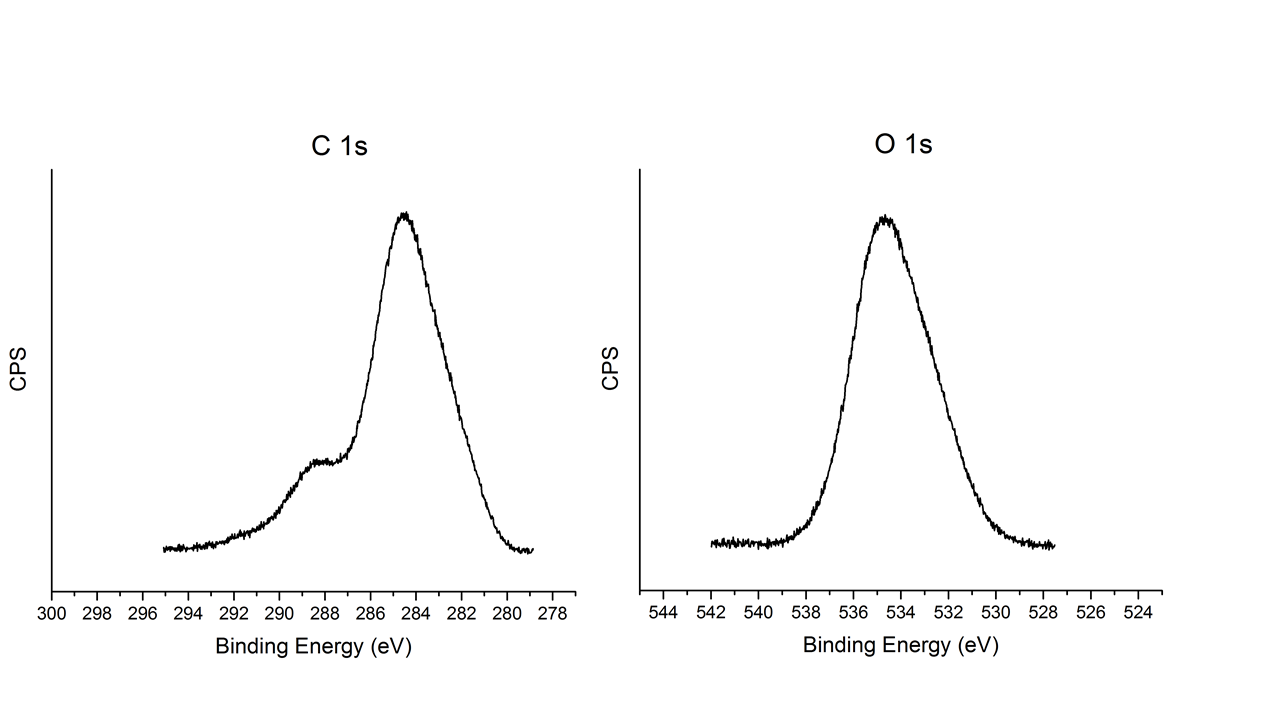


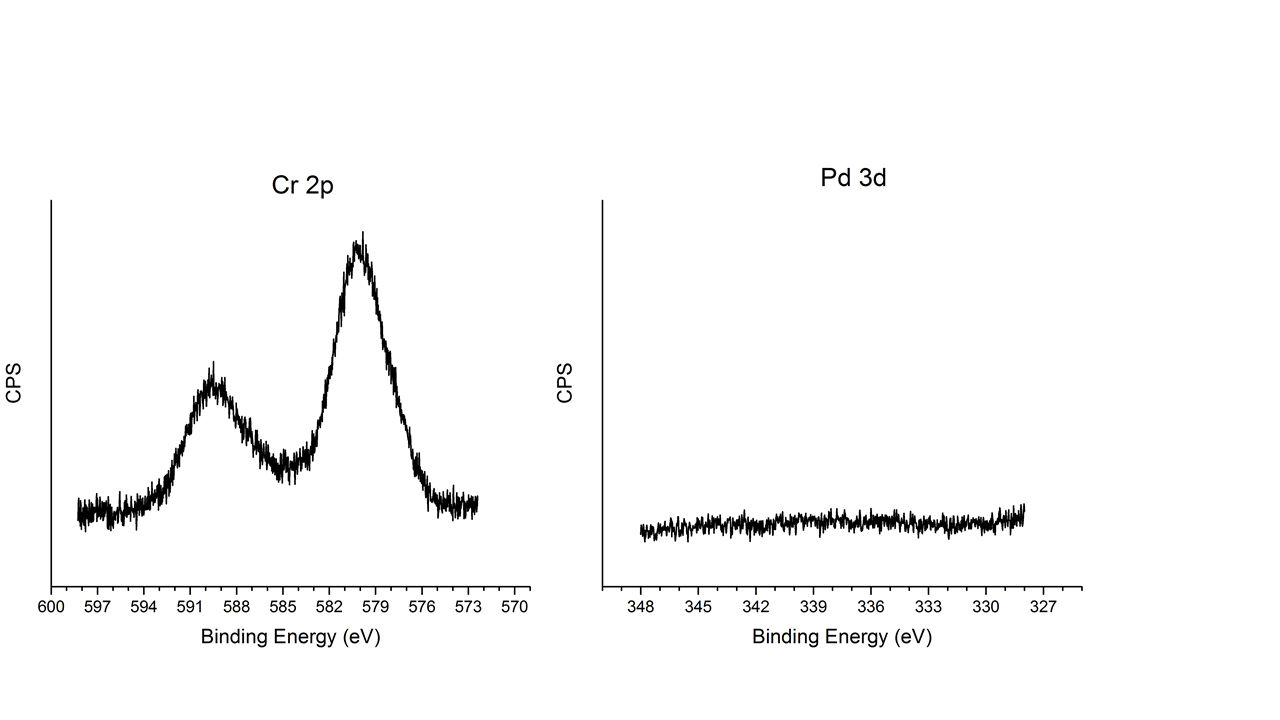


**Figure S4** XPS of 1.0 wt% Pd MIL-101(Cr)


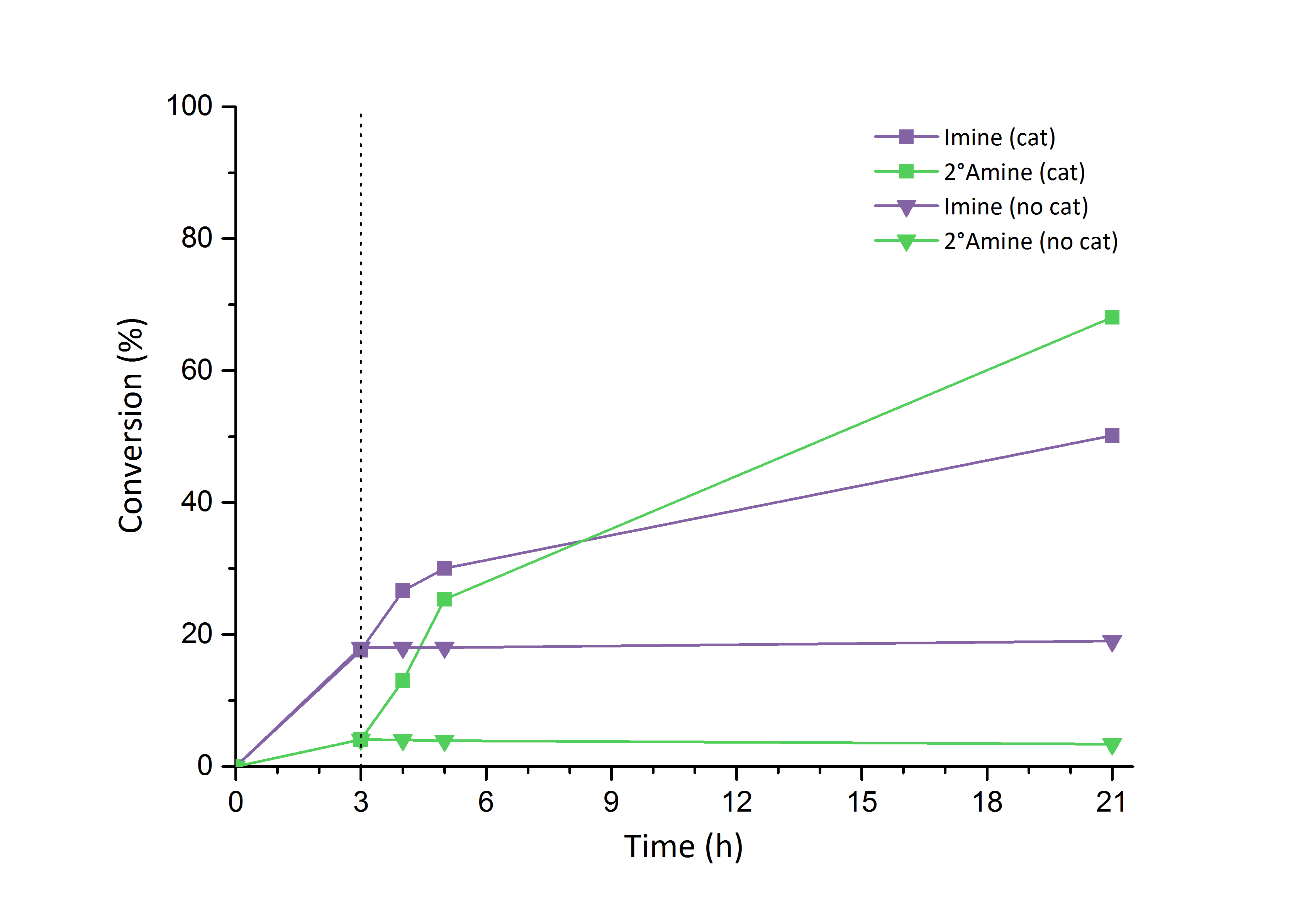


**Figure S5** Hot filtration test on 1.0 wt% Pd MIL-101(Cr) at 50 °C. The vertical dotted line at 3 hours represents the time which the catalyst was removed from half of the reaction vials.


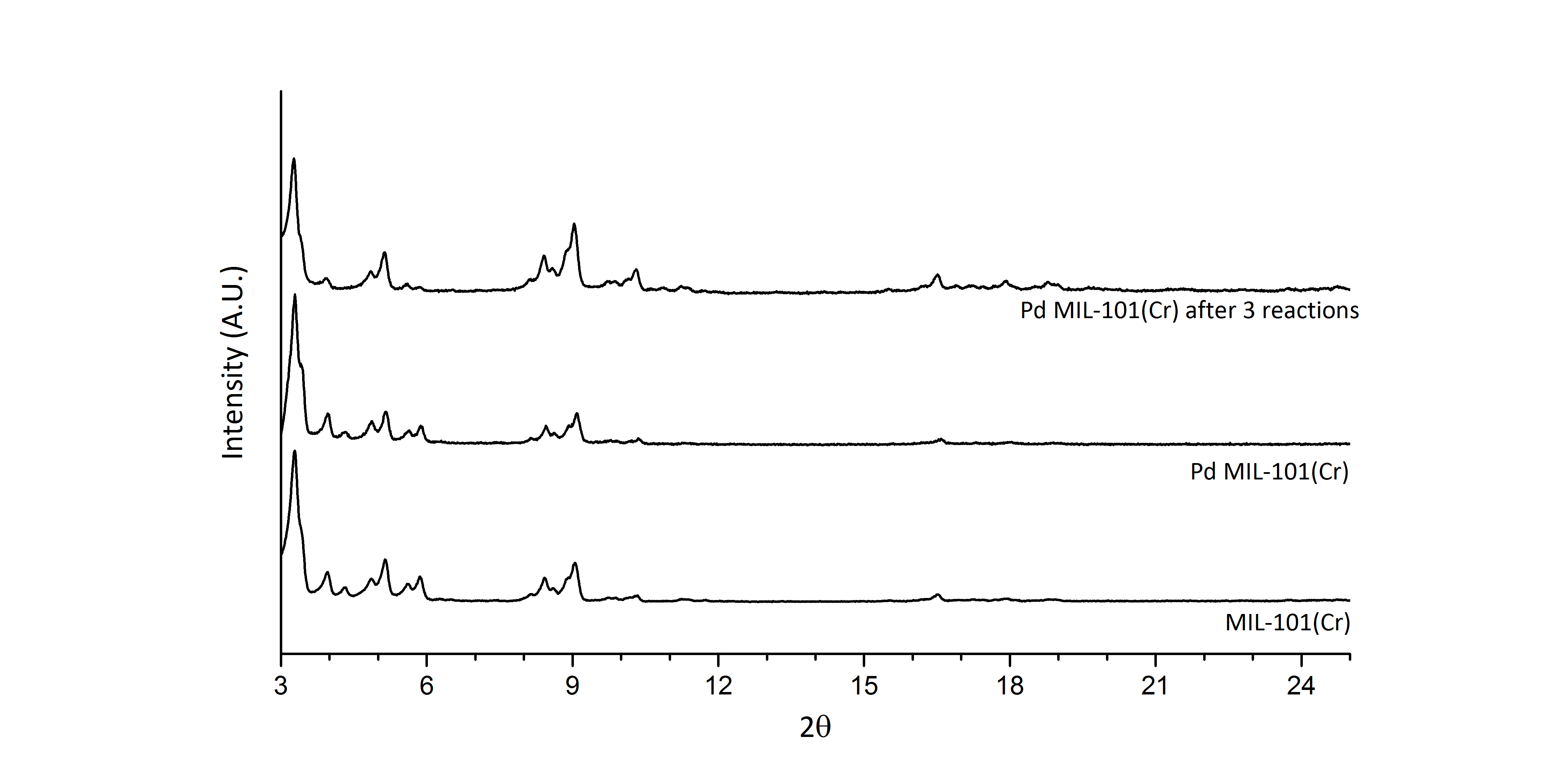


**Figure S6** Pd MIL-101(Cr) XRD patterns before and after recycling experiments at 50 °C.


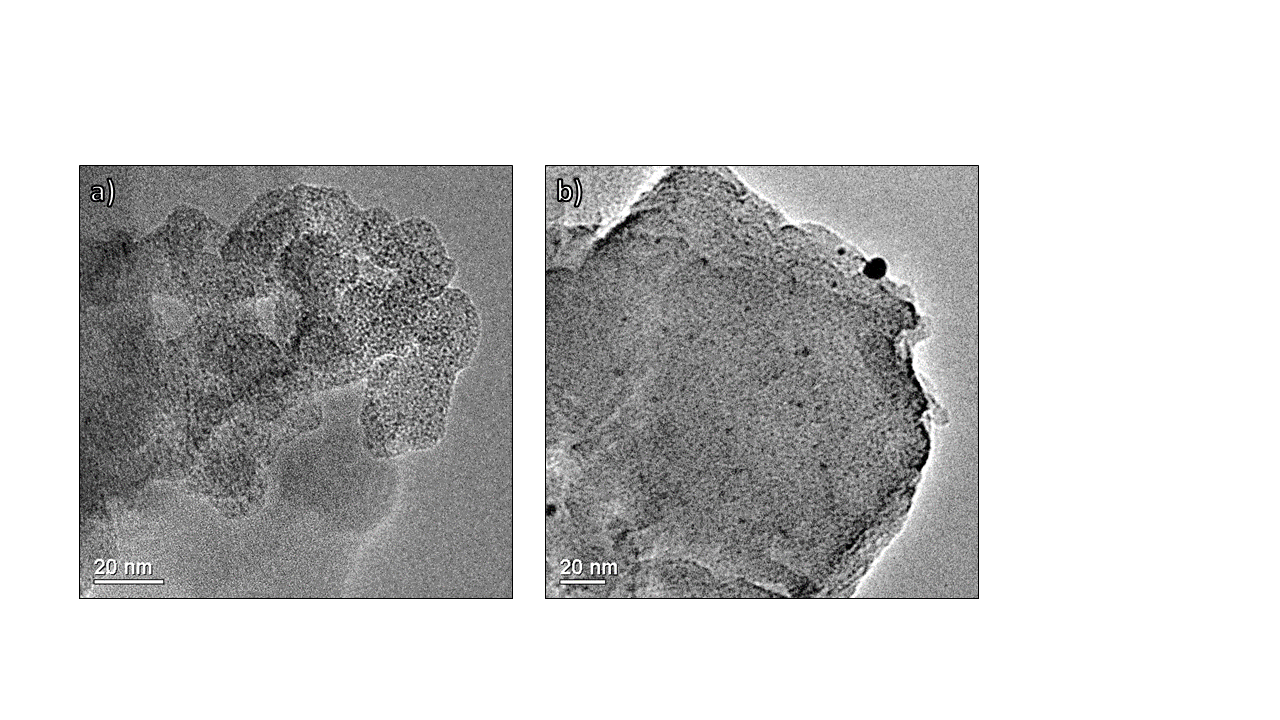


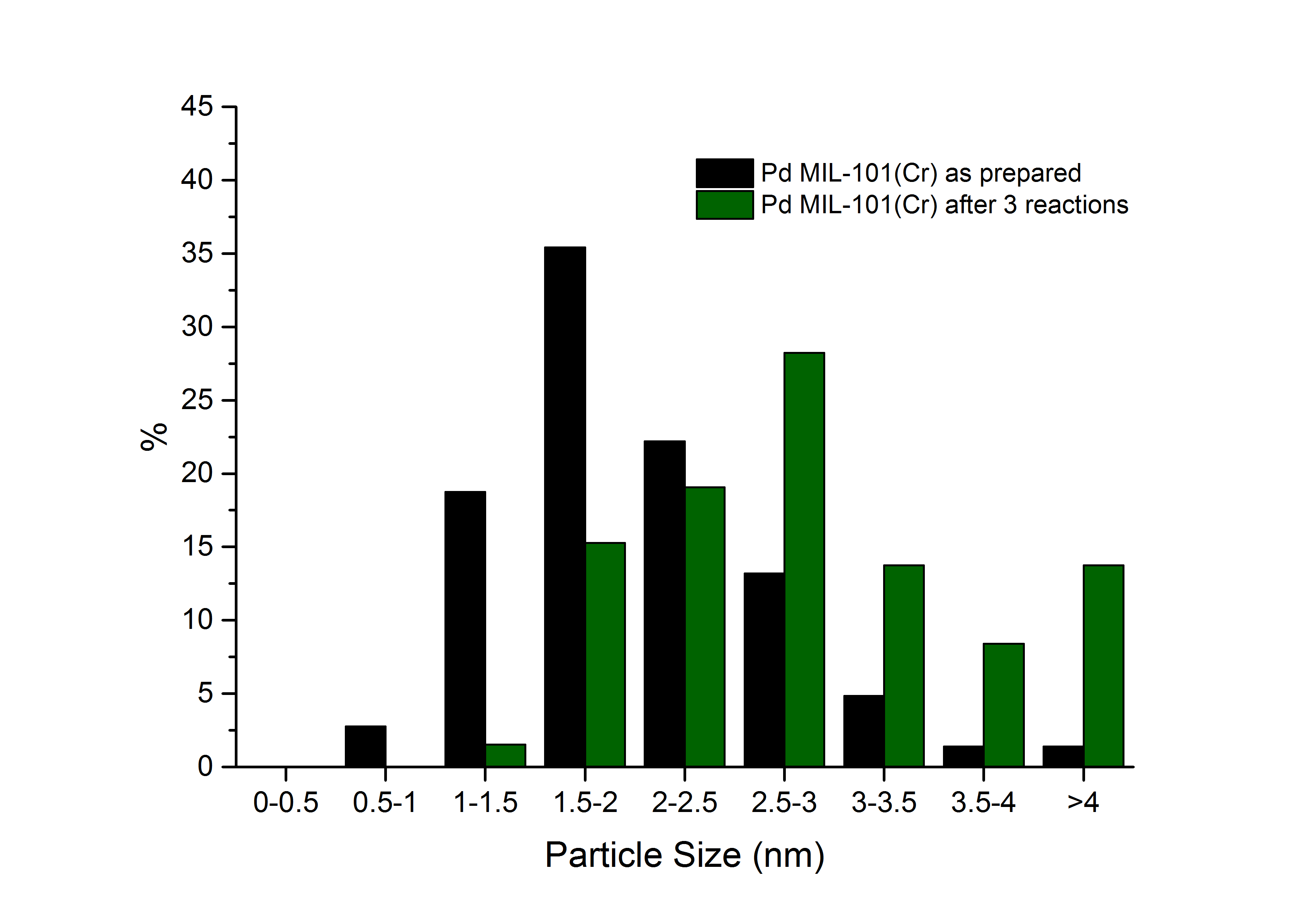


**Figure S7** (Above) Pd MIL-101(Cr) (a) as prepared and (b) after 3 catalysis runs at 50 °C; (below) Pd nanoparticle size distribution of as-prepared and reused 1 wt% Pd@MIL-101(Cr) at 50 °C

Heterogeneity of Catalysis, Re-usability and Recyclability at 90 °C


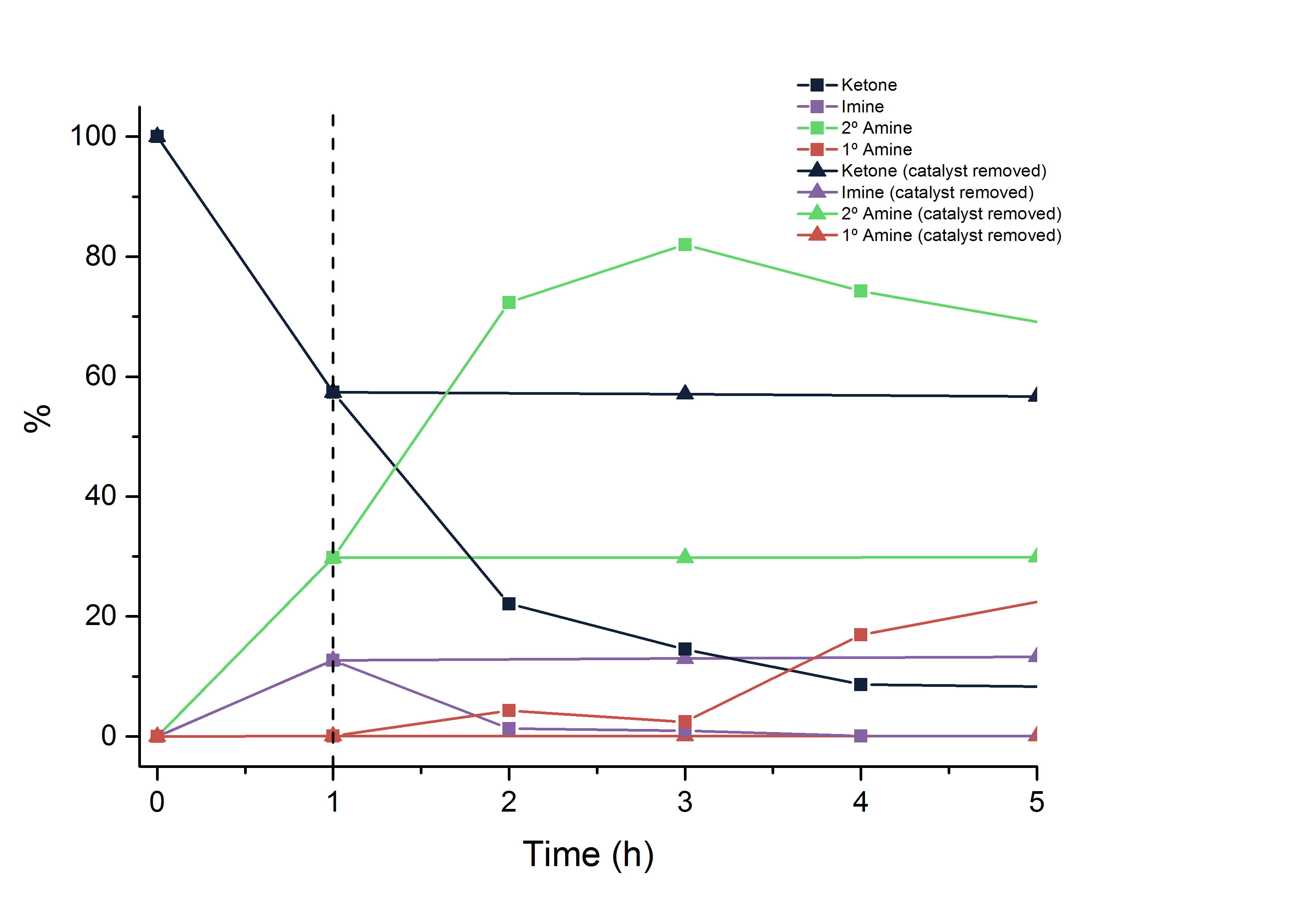


**Figure S8** Hot filtration test on 1.0 wt% Pd MIL-101(Cr) at 90 °C. Squares are the reaction progressing with the catalyst and triangles are the reaction proceeding after the catalyst has been removed at 1 h.

**
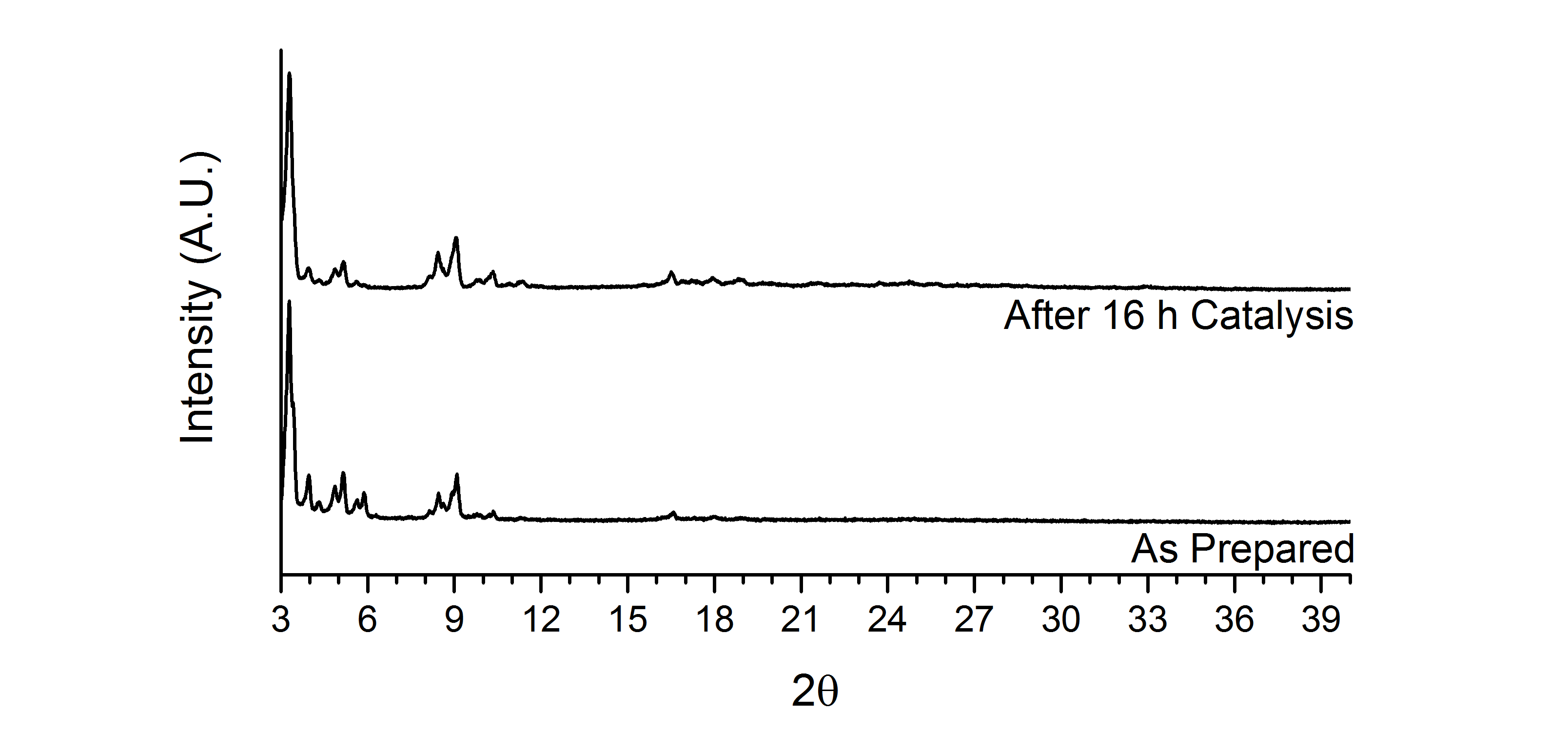
**

**Figure S9** shows the 1.0 wt% Pd MIL-101(Cr) material remains crystalline after catalysis.


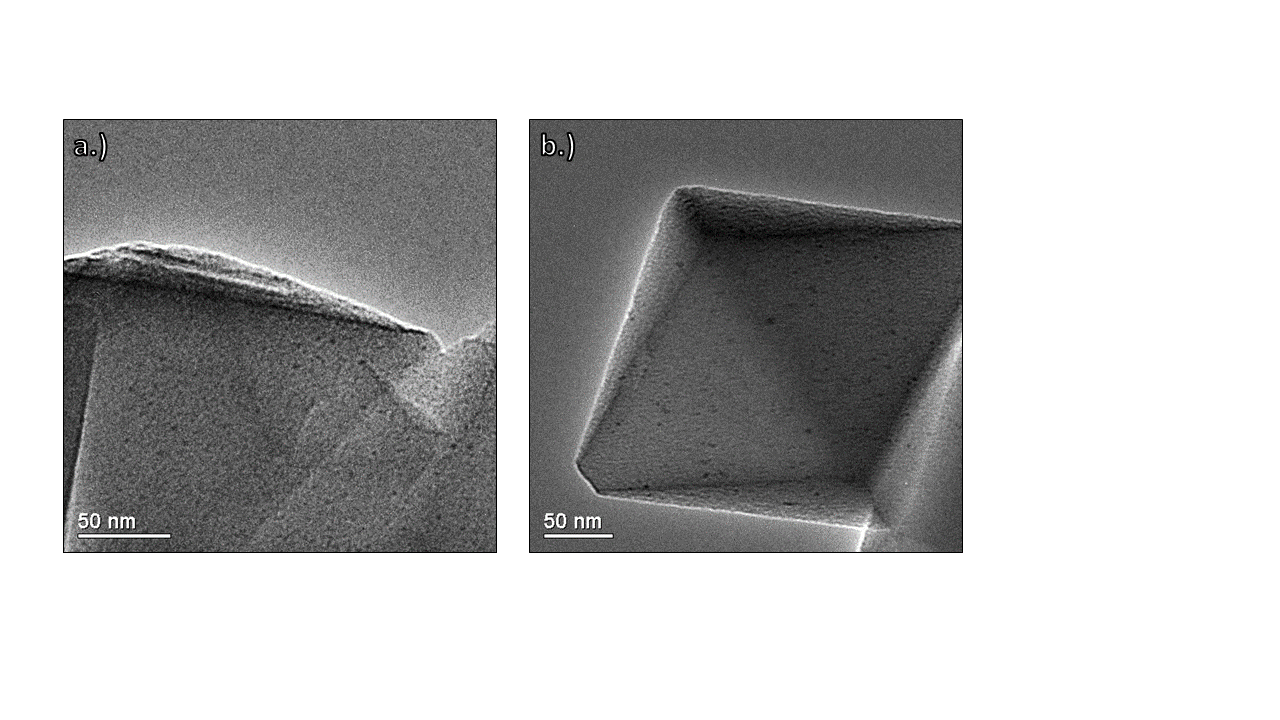


**Figure S10** TEM of (left) as-prepared 1.0 wt% Pd@MIL-101(Cr) and (right) the same material after catalysis at 90 °C for 16 h.

Modelling Imine Formation

The imine formation reaction was performed at varying reaction times using MIL-101(Cr) at 90 °C and 10 bar H_2_. The data were then used to calculate and determine the forward and reverse rate constants [1]. Two sets of rate constants were calculated: with and without water as a variable in the equations. This means that with water the reaction is second order in both directions and without water the reaction is second order in the formation of the imine and first order in the reverse reaction. Boeker defined these equations for reversible bimolecular reactions such that the rate constants are easily solvable [1]. These equations are shown in the Equations 1 with water and 7 without water. Using these equations and plotting Z (Equation 3) against time gives a straight line with a slope of m (Equation 4). Using the slope, it is possible to determine the reaction rate constants for both reactions shown in Equations 5 and 6 with water and Equations 11 and 12 without water. Within these equations, concentrations subscripted “e” indicate the concentration at equilibrium, and concentrations subscripted with “0” indicate the initial concentration.

|  | $A+B\leftrightarrow I+W$ |  |
| --- | --- | --- |
|  | $\left[ k_{1}\left( 1-\frac{1}{K_{e}} \right) \right]\left[ \frac{K_{e}\left( A_{e}+B_{e} \right)+I_{e}+W_{e}}{\left( K_{e}-1 \right)} \right]t=$  $-\ln\left( 1-\frac{I-I_{0}}{I_{e}-I_{0}} \right)+\ln\left( 1-\frac{I-I_{0}}{\left[ \frac{K_{e}\left( A_{e}+B_{e} \right)+I_{e}+W_{e}}{\left( K_{e}-1 \right)} \right]+I_{e}-I_{0}} \right)$ | 1 |
|  | $K_{e}=\frac{I_{e}W_{e}}{A_{e}B_{e}}=\frac{k_{1}}{k_{1,r}}$ | 2 |
|  | $Z= -\ln\left( 1-\frac{I-I_{0}}{I_{e}-I_{0}} \right)+\ln\left( 1-\frac{I-I_{0}}{\left[ \frac{K_{e}\left( A_{e}+B_{e} \right)+I_{e}+W_{e}}{\left( K_{e}-1 \right)} \right]+I_{e}-I_{0}} \right)$ | 3 |
|  | $m= \left[ k_{1}\left( 1-\frac{1}{K_{e}} \right) \right]\left[ \frac{K_{e}\left( A_{e}+B_{e} \right)+I_{e}+W_{e}}{\left( K_{e}-1 \right)} \right]$ | 4 |
|  | $k_{1}=\frac{m}{\left( 1-\frac{1}{K_{e}} \right)\left[ \frac{K_{e}\left( A_{e}+B_{e} \right)+I_{e}+W_{e}}{\left( K_{e}-1 \right)} \right]}$ | 5 |
|  | $k_{1,r}=\frac{k_{1}}{K_{e}}$ | 6 |
|  |  |  |
|  | $A+B\leftrightarrow I$ |  |
|  | $k_{1}\left[ \frac{K_{e}\left( A_{e}+B_{e} \right)+I_{e}}{\left( K_{e}-1 \right)} \right]t=$  $-\ln\left( 1-\frac{I-I_{0}}{I_{e}-I_{0}} \right)+\ln\left( 1-\frac{I-I_{0}}{\left[ \frac{A_{e}+B_{e}+1}{K_{e}} \right]+I_{e}-I_{0}} \right)$ | 7 |
|  | $K_{e}=\frac{I_{e}}{A_{e}B_{e}}=\frac{k_{1}}{k_{1,r}}$ | 8 |
|  | $Z=-\ln\left( 1-\frac{I-I_{0}}{I_{e}-I_{0}} \right)+\ln\left( 1-\frac{I-I_{0}}{\left[ \frac{A_{e}+B_{e}+1}{K_{e}} \right]+I_{e}-I_{0}} \right)$ | 9 |
|  | $m=k_{1}\left[ \frac{K_{e}\left( A_{e}+B_{e} \right)+I_{e}}{\left( K_{e}-1 \right)} \right]$ | 10 |
|  | $k_{1}=\frac{m}{\left[ \frac{K_{e}\left( A_{e}+B_{e} \right)+I_{e}}{\left( K_{e}-1 \right)} \right]}$ | 11 |
|  | $k_{1,r}=\frac{k_{1}}{K_{e}}$ | 12 |
|  |  |  |
|  | $\frac{dC_{A}}{dt}={-k}_{1}C_{A}C_{B}+k_{1,r}C_{I}C_{W}$ | 13 |
|  | $\frac{dC_{A}}{dt}={-k}_{1}C_{A}C_{B}+k_{1,r}C_{I}$ | 14 |

From the calculations, the reaction rate constants were added into MATLAB programs using Equations 13 or 14. The models and experimental data are shown in Figure S13. The calculated rate constants when water is taken in account are k_1_ = 2.03 M^-1^ h^-1^ and k_1,r_ = 0.76 M^-1^ h^-1^. The calculated rate constants when water was not included are k_1_ = 2.1 M^-1^ h^-1^ and k_1,r_ = 0.03 h^-1^. There is almost no difference in the water being present, so the simpler model with a first order reverse reaction was used throughout.

[1] Boeker, E. A., Simple Integrated Rate Equations for Reversible Bimolecular Reactions. *Experientia* **1984,** *40*, 453-456.


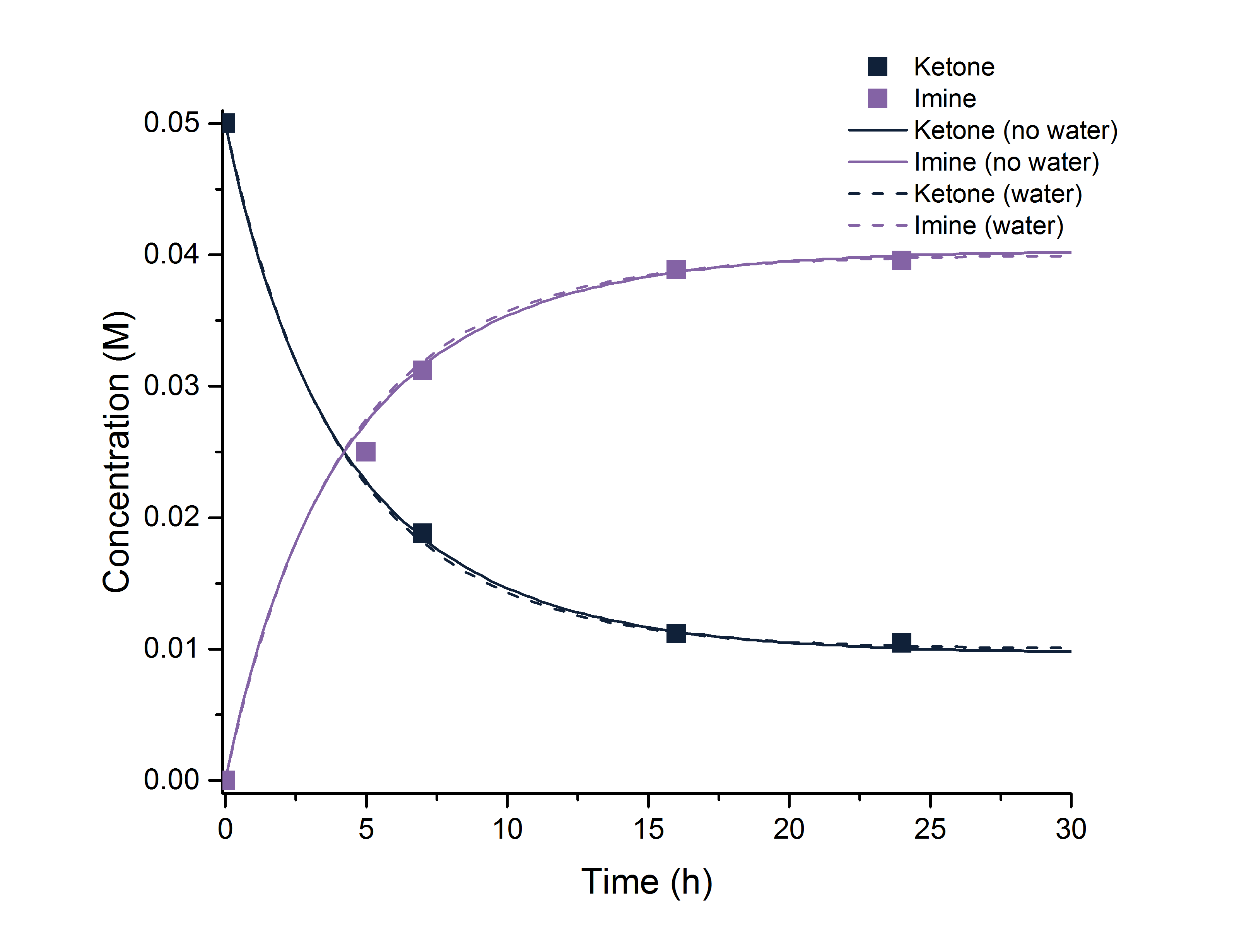


**Figure S11**  Reversible imine formation with reaction rate constants including water in the reverse step (k_1_ = 2.03 M^-1^ h^-1^, k_1,r_ = 0.76 M^-1^ h^-1^) and not including water (k_1_ = 2.1 M^-1^ h^-1^, k_1,r_ = 0.03 h^-1^).

**Table S2** Imine formation (equal to ketone conversion) over MIL-101(Cr) with and without 1 wt% Pd at 90 °C under different gas environments

| Entry | Catalyst | Catal. amt./ mol% | Pressure | Time / h | Conversion / % |
| --- | --- | --- | --- | --- | --- |
| 1 | MIL-101(Cr) | 5 | 10 bar H_2_ | 7 | 62 |
| 2 | MIL-101(Cr) | 10 | 10 bar Ar | 7 | 36 |
| 3 | Pd@MIL-101(Cr) | 5 | 10 bar Ar | 7 | 50 |
| 4 | MIL-101(Cr) | 5 | 10 bar H_2_ | 16 | 78 |
| 5 | MIL-101(Cr) | 10 | 10 bar Ar | 16 | 52 |
| 6 | Pd@MIL-101(Cr) | 5 | 10 bar Ar | 16 | 66 |
